# Change in Pseudomonas *aeruginosa* prevalence in cystic fibrosis adults over time

**DOI:** 10.1186/s12890-016-0333-y

**Published:** 2016-12-07

**Authors:** Mathew R. Crull, Kathleen J. Ramos, Ellen Caldwell, Nicole Mayer-Hamblett, Moira L. Aitken, Christopher H. Goss

**Affiliations:** 1 Department of Medicine, University of Washington, Seattle, WA USA; 2 Department of Pediatrics, Division of Pulmonary, University of Washington, Seattle, WA USA; 3 Department of Biostatistics, University of Washington, Seattle, WA USA; 4 University of Washington Medical Center, Campus Box 356522, 1959 N.E. Pacific, Seattle, WA 98195 USA

**Keywords:** Cystic fibrosis, Pseudomonas aeruginosa, Chronic infection, Epidemiology, Temporal trends

## Abstract

**Background:**

Little is known about risk factors for chronic and mucoid *Pseudomonas aeruginosa* (Pa) infection in cystic fibrosis (CF) adults, and whether the prevalence is changing.

**Methods:**

We employed a retrospective cohort to analyze data from a single adult CF center (2002 to 2012). Regression models were used to assess independent predictors and change in prevalence of chronic and mucoid Pa infection over time.

**Results:**

The odds ratio of mucoid Pa infection was significantly less in individuals with better baseline lung function (OR 0.84,95%CI:0.77–0.92) and those diagnosed after the age of 25 (OR 0.21, 95%CI:0.05–0.95). The prevalence of chronic Pa and mucoid Pa decreased during the time interval. After adjusting for confounders, the observed decrease in chronic and mucoid Pa between 2002 and 2012 was no longer significant.

**Conclusions:**

The prevalence of chronic and mucoid Pa is decreasing. Larger studies are needed to confirm these regional trends and their significance.

**Electronic supplementary material:**

The online version of this article (doi:10.1186/s12890-016-0333-y) contains supplementary material, which is available to authorized users.

## Background

Cystic Fibrosis (CF) is the most common severe monogenic disorder among Caucasians [1]. CF was once considered a fatal childhood disease, but with advances in care, survival has steadily improved. Mortality decreased by 1.8% per year between the years 2000 to 2012, and if this observed rate continues, individuals born with CF in 2010 are predicted to have a median life expectancy of more than 50 years [2]. In 2013, half of the US CF population was 18 years of age or older [3]. Chronic lung disease in CF, however, continues to cause significant morbidity, and respiratory failure is the leading cause of death in this population [4]. Chronic endobronchial infections are central in the pathogenesis of chronic lung disease [5].

*Pseudomonas aeruginosa* (Pa) is the most common cultured respiratory pathogen in CF, and in children, Pa is associated with a more rapid decline in lung function and worse survival [6–8]. Prior reports estimated that by age eighteen, 80% of individuals with CF are infected with Pa [9]. Over time, most individuals with CF become chronically infected with Pa, and a mucoid phenotype of Pa frequently becomes the predominant form found in culture [10, 11]. Chronic and mucoid Pa infections are associated with increased morbidity and mortality when compared to individuals not infected with Pa [12, 13]. Additionally, due to formation of biofilms, development of antibiotic resistance and overall bacterial abundance, this form of Pa infection is challenging to eradicate [5, 14, 15].

Because of the challenges with eradicating chronic and mucoid Pa, the focus of treatment has been on preventing the chronic infection [16]. Recent studies in children demonstrated eradication of newly acquired Pa from airway is associated with delayed reacquisition of Pa even after stopping antibiotics [17–20]. These studies suggest that development of chronic Pa infection might be decreasing, but this has never been formally investigated in the adult CF population. Using the CF Foundation Patient Registry a recent study showed a decline in both the incidence and prevalence of Pa between 2006 and 2012 [21]. This study, however, did not look specifically at chronic or mucoid Pa infection.

We hypothesize the prevalence of chronic and mucoid Pa lung infection in the older CF population is decreasing with time and there are independent predictors of chronic and mucoid Pa infection in CF adults, which may be unique compared to the younger CF population. Using a longitudinal adult CF cohort, we sought to assess the change in both chronic and mucoid Pa infections between 2002 and 2012 and identify independent predictors of the chronically infected and mucoid Pa states.

## Methods

### Population

A retrospective cohort study design was employed using individuals age 18 or older receiving care through the Adult CF Clinic at the University of Washington Medical Center between 2002 and 2012. Patients were able to contribute data from multiple years; however, all individuals with no evaluable culture data during a 1-year period were excluded for the calendar year in which the data was absent. No imputation of missing data was performed. Sensitivity analyses were performed to assess the impact of missing data.

### Definitions of predictors of interest

Both time independent and time dependent variables were considered as potential independent predictors of chronic and mucoid Pa. Time independent variables included age at diagnosis, gender, CF mutation status, CF transmembrane conductance regulator (CFTR) function and baseline lung function. Time dependent covariates included age, CF-related diabetes (CFRD) and pancreatic insufficiency (PI). Age at diagnosis was examined both as a continuous and binary variable. As a binary variable, age at diagnosis was categorized as 25 years of age or younger and greater than 25 years of age. CFTR function was classified as minimal (class I, II, and III), and residual (class IV and V). All remaining genotypes were grouped together along with those with unknown mutations. The residual CFTR function group was used as the comparator group. CFRD was defined by receiving insulin, and PI was defined as receiving pancreatic enzymes. Percent predicted forced expiratory volume in one second (FEV_1_) at time of cohort entry was used to define baseline lung function. When analyzing the change in prevalence of both chronic Pa and mucoid Pa, potential confounding variables included age, age of diagnosis, gender, CFTR function, and baseline lung function. The inclusion of confounding variables was made a priori.

Anticipating relatively small numbers of individuals within each year, calendar time was divided into four time periods (2002–2004, 2005–2007, 2008–2010, and 2011–2012) for analysis of change in prevalence. This decision was made prior to analysis of the data. This division of the observation period balanced the number of subjects within each time period.

### Outcome definitions

The primary outcomes of interest for identification of risk factors and change in prevalence were chronic Pa and mucoid Pa infection. Due to the frequency of respiratory culturing in this cohort, chronic Pa infection was defined using a modified definition of chronic Pa initially proposed by Lee et al. [22]. Individuals were defined as chronic when three or more respiratory cultures were provided in a calendar year and 50% or more were positive for Pa. If only two cultures were obtained in a calendar year, both cultures needed to be positive for Pa to be considered chronically infected. Those individuals who provided less than 2 cultures within a calendar year were excluded from the primary analysis. A sensitivity analysis was performed including all subjects regardless of the number of cultures provided within a year. Mucoid Pa was defined having at least one respiratory culture with the mucoid phenotype during a calendar year. Sensitivity analyses looking at the influence of CFTR function, duration of time periods, changes in cohort characteristics and inhaled antibiotic use were also performed.

### Analysis

Descriptive statistics were used to describe the cohort. Continuous variables were reported as medians with inter quartile ranges and categorical variables were reported as proportions. To account for repeated measurements, population averaged generalized estimating equation (GEE) regression models with independent working correlation were used to assess independent predictors and change in prevalence of both chronic and mucoid Pa infection over time [23]. When assessing independent predictors of chronic and mucoid Pa infection, a univariate regression model was first used to screen for significant variables. Those variables with a significance level less than or equal to 0.1 were then included in a multivariable regression model that included an indicator for time period to account for changes in cohort over the observation period. Both univariate and multivariable regression analysis were used when assessing the change in prevalence of chronic and mucoid Pa over time. Odds ratio with a 95% confidence interval was used to report results of the GEE models. Unless otherwise noted, a two-tailed P-value, not adjusted for multiple comparisons, less than 0.05 was considered statistically significant. All analyses were performed using STATA version 13 (StataCorp LP, College Station, Texas). The University of Washington Institutional Review Board approved this study (45798).

## Results

### Overview of cohort

During the observation period, data was collected from 402 individuals. The average observation period was 4.6 years (range 1–11 years). Demographic data for the cohort from the year of entry is summarized in Table 1. This cohort was dynamic with individuals aging or migrating into the population throughout the observation period. Additionally, some individuals left the cohort due to death or out-migration. Changes in population characteristics during the observation period are summarized in Additional file 1: Table E1. The cohort increased from 123 patients in 2002 to 211 patients in 2012. Genotype data was available on 382 individuals (95%) in the cohort. Over the 11 years, the proportion of individuals homozygous for f508del decreased from 57.7 to 48.8%. Mean population percent-predicted FEV_1_ increased from 59.0 to 68.0% of predicted. The proportion using an inhaled anti-pseudomonal antibiotic remained relatively constant during the observation period. A cross sectional comparison of cohort demographics between 2002 and 2012 based on state of Pa infection and number of cultures is presented in Additional file 1: Table E2 and E3 respectively.

**Table 1:** Characteristics of cohort at time of entry into the cohort

	(*N* = 402)
Mean age, years (SD)	29.7 (9.4)
Median age of diagnosis, years (IQR)	1.5 (8)
Gender, % Female (N)	48.8 (196)
Race, % Caucasian (N)	94.3 (379)
Ethnicity, % Hispanic (N)	1.9 (7)
f508del status	
Homozygous, % (N)	52.4 (200)
Heterozygous, % (N)	39.0 (149)
Other, % (N)	8.6 (33)
CFTR Mutation Classification ^ad^	
Minimal, % (N)	71.7 (274)
Residual, % (N)	10.7 (41)
Unclassified, % (N)	17.5 (67)
FEV_1_, % predicted (SD)^d^	63.5 (26.0)
Co-morbidities	
CF-related diabetes ^bd^, % (N)	28.0 (109)
Pancreatic insufficiency ^cd^, % (N)	86.3 (308)
Inhaled Antibiotic Use^d^	49.5 (147)
Microbiology	
Any form of Pseudomonas isolated, % (N)	64.2 (238)
Mucoid Pseudomonas phenotype, % (N)	48.0 (178)

*SD* standard deviation, *IQR* inter quartile range

^a^Minimal: Both alleles containing mutations resulting in minimal CFTR function (Class 1, 2, or 3); Residual: at least one allele containing mutation resulting in partial CFTR function (Class 4 or 5); Unclassified: at least one allele with unknown CFTR function and if other allele function known, mutation resulting in minimal CFTR function

^b^CF-related diabetes: use of insulin

^c^Pancreatic insufficiency: use of pancreatic enzymes

^d^Missing data (N, %): CFTR Mutation Classification (20, 5.0%), FEV_1_ (47, 11.7%), CFRD (12, 3.0%), PI (45, 11.2%), Inhaled antibiotic use (105, 26%)

### Risk factors for chronic and mucoid Pa

Results of the univariate and multivariable analysis for independent predictors of chronic Pa infection using a modified Leeds definition and mucoid Pa are in Table 2. Subjects with worse baseline lung function were significantly more likely to have mucoid Pa (*P*-value < 0.001). When comparing two groups with a 10% difference in baseline lung function, the odds of having a mucoid Pa infection was 16% less in the group with better lung function (OR: 0.84, 95% CI: 0.77–0.92). Those individuals diagnosed after the age of 25 were less likely to have a mucoid Pa infection (OR: 0.21, 95% CI: 0.05–0.95). All other variables included in both the mucoid Pa and chronic Pa multivariable models did not reach a significance level less than 0.05 (Table 2).

**Table 2:** Independent predictors of chronic and mucoid Pa infection

	Chronic Pa	Mucoid Pa
	OR (95%CI)	OR (95%CI)
Univariate		
Age (yr)	0.83 (0.63–1.10)	0.82 (0.66–1.02)
Diagnosis		
Age (yr)	0.96 (0.94–0.98)	0.97 (0.96–0.99)
Diagnosis after 25 years of age	0.12 (0.04–0.31)	0.21 (0.09–0.46)
Gender, female	1.07 (0.65–1.77)	0.94 (0.61–1.44)
CFTR function^a^	1.94 (1.10–3.44)	2.61 (1.28–5.63)
CF-related diabetes^b^	1.02 (0.63–1.65)	0.88 (0.57–1.34)
Pancreatic insufficiency^c^	2.21 (1.11–4.37)	2.52 (1.46–4.38)
Lung Function^d^	0.90 (0.82–1.00)	0.79 (0.71–0.88)
Multivariable^e^		
Age (yr)	1.03 (0.99–1.08)	1.00 (0.97–1.03)
Diagnosis		
Age (yr)	0.96 (0.91–1.02)	1.01 (0.97–1.05)
Diagnosis after 25 years of age	0.18 (0.03–1.20)	0.21 (0.05-0.95)
CFTR function^a^	0.51 (0.17–1.55)	0.99 (0.42-2.34)
Pancreatic insufficiency^c^	1.23 (0.53–2.82)	1.58 (0.89–2.80)
Lung Function^d^	0.91 (0.82–1.01)	0.84 (0.77–0.92)

^a^Comparator group: Residual CFTR function: at least one allele containing mutation resulting in partial CFTR function (Class 4 or 5)

^b^CF-related diabetes: use of insulin

^c^Pancreatic insufficiency: use of pancreatic enzymes

^d^10% difference in percent predicted FEV_1_

^e^Variables with significance level ≤ 0.1 included in multivariable analysis

### Prevalence of chronic Pa over time

**Fig. 1:**
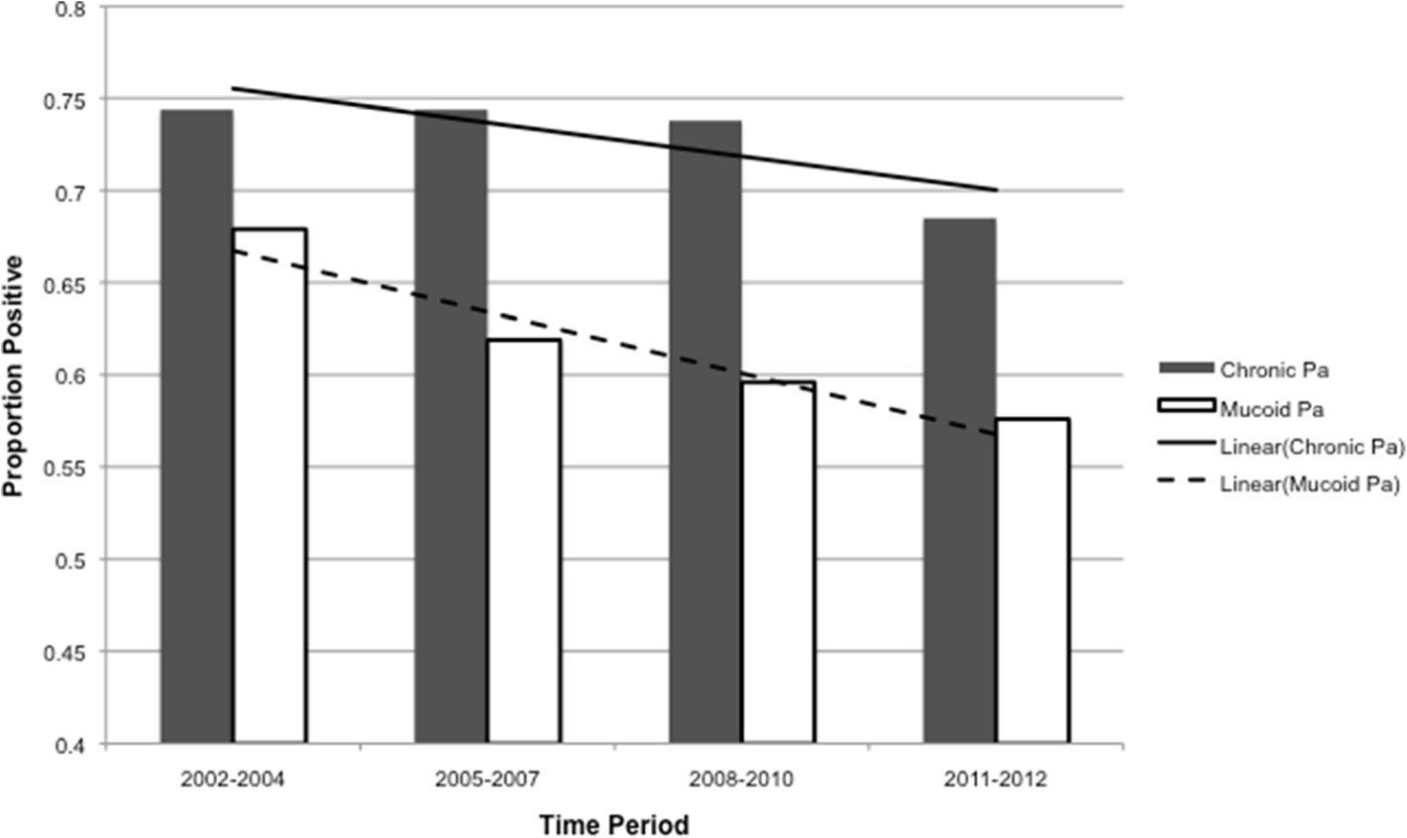
By time period, prevalence of chronic Pa by modified Leeds Criteria and mucoid Pa

The prevalence of chronic Pa in those who provided at least two respiratory cultures in a calendar year decreased from 73.5% in 2002 to 67.0% in 2012 (Additional file 1: Table E1). The average prevalence of chronic Pa infection decreased by 5.9% from the 2002 to 2004 time period to the 2011 to 2012 time period (Fig. 1). After adjusting for gender, age, age at diagnosis, CFTR function and baseline lung function, the odds ratio of chronic Pa infection in the 2011–2012 time period relative to 2002–2004 time period was 0.82 (95% CI: 0.31–2.14) (Table 3). Relative to the 2002–2004 time period, odds ratio of chronic Pa infection was not significantly different than other time periods. In both the unadjusted and adjusted models of chronic Pseudomonas infection, the P-values of the trend test for time period were not significant (Test of trend: unadjusted *P* = 0.25; adjusted *P* = 0.84) (Table 3).

**Table 3:** Odds ratio for prevalence of chronic *Pseudomonas aeruginosa* infection using modified Leeds criteria

		Adjusted
Year	OR^a^ (95% CI)	OR^b,c^ (95% CI)
2002–2004	-	-
2005–2007	1.00 (0.65–1.53)	1.22 (0.42–3.52)
2008–2010	0.97 (0.60–1.56)	1.29 (0.55–3.00)
2011–2012	0.75 (0.46–1.21)	0.82 (0.31–2.14)

^a^Trend test p: 0.25

^b^Trend test p: 0.85

^c^Adjusted for age, gender, age at diagnosis, CFTR function (minimal: class 1, 2, 3; residual: class 4, 5; Unclassified), baseline lung function (percent predicted FEV1)

### Prevalence of mucoid Pa over time

The prevalence of isolating the mucoid phenotype of Pa on respiratory culture in 2002 was 68.2% and in 2012 the prevalence was 58.1% (Additional file 1: Table E1). The mean prevalence of mucoid Pa decreased by 10.3% when comparing the 2002–2004 time period and the 2011–2012 time period (Fig. 1). When comparing the 2011–2012 time period to the 2002–2004 time period, the odds ratio of isolating the mucoid Pa phenotype in culture was 0.64 (95% CI: 0.43–0.96) with a significant test of trend (*p* = 0.04), but after adjusting for potential confounding the change was no longer significant (OR 0.55; 95% CI: 0.27–1.11) (Table 4). Demographic data was similar between the chronic Pa by modified Leeds definition and the mucoid Pa phenotype group in 2002 and 2012 (Additional file 1: Table E4).

**Table 4:** Odds ratio for prevalence of mucoid *Pseudomonas aeruginosa* phenotype in culture

		Adjusted
Year	OR^a^(95% CI)	OR^b,c^ (95% CI)
2002–2004	-	-
2005–2007	0.77 (0.56–1.06)	0.81 (0.40–1.67)
2008–2010	0.70 (0.57–1.03)	0.77 (0.39–1.52)
2011–2012	0.64 (0.43–0.96)	0.55 (0.27–1.11)

^a^Trend test p: 0.04

^b^Trend test p: 0.11

^c^Adjusted for age, gender, age at diagnosis, CFTR function (minimal: class 1, 2, 3; residual: class 4, 5; Unclassified), baseline lung function (percent predicted FEV_1_)

### Sensitivity analysis

When restricting the cohort to only individuals homozygous for the f508del mutation, a trend toward decreasing prevalence of both chronic and mucoid Pa was observed (Additional file 1: Table E5). This trend was not significant for either group. Inclusion of all individuals who provided less two respiratory cultures within a year as free of chronic infection resulted in a significant decrease in the prevalence of chronic Pa infection (Test of trend P-value 0.002) (Additional file 1: Table E6). Adjusting for inhaled antibiotic use did not significantly change the observed trend in prevalence of chronic or mucoid Pa infection (Additional file 1: Table E7). Using a two time period model (2002–2007, 2008–2012), the odds of infection was decreased but not significantly in the later time period relative to the early period for both chronic (OR 0.86; 95% CI: 0.62–1.21) and mucoid (0.78; 95% CI: 0.59–1.02) Pa infections.

## Discussion

Respiratory failure is the leading cause of death in CF, and Pa infection, particularly chronic and the mucoid phenotype, has been associated with increased morbidity and mortality [7, 10, 12, 13]. Using a single center adult CF cohort, we observed a decrease in prevalence of both chronic and mucoid Pa over an 11-year observation period. In a multivariable model adjusting for potential confounding and repeated usage of patients, the change in prevalence of both chronic and mucoid Pa, however, did not meet the pre-specified significance level of 0.05. We found age of diagnosis, CFTR function, PI and lung function to be predictors of both chronic and mucoid Pa when using a univariate analysis. Age of diagnosis and baseline lung function remained significant independent predictors of mucoid Pa infection after adjusting our analysis for potential confounding. This suggests that in the adult CF population individuals are less likely to have a mucoid Pa infection when they are either diagnosed at a later age or have preserved lung function.

While our observed change in Pa over time may be due to chance, a possible explanation for the decreasing prevalence over time is a change in the population characteristics of this cohort. The hypothesis is supported when looking at the change in mucoid Pa prevalence during the observation period. A significant change in prevalence over time was observed in the unadjusted analysis of mucoid Pa. This association was no longer significant after adjusting for clinical and demographic variables that may confound the association between time and change in prevalence. Comparing all individuals making up the cohort in 2002 to 2012, the proportion of individuals in the cohort homozygous for f508del decreased. Along with this change there was an increase in the proportion of individuals with an unknown CFTR functional class. With genetic testing, the proportion of adults diagnosed with CF has increased, and of these newly diagnosed adults, fewer are homozygous for f508del [24]. Residual CFTR function may result in less chronic infection. A recent study demonstrated a decrease in the prevalence of Pa in respiratory cultures in a group of individuals with the G551D mutation receiving ivacaftor. CFTR having a role in innate host response to infection was a postulated explanation [25]. While CFTR function was not associated with chronic or mucoid Pa infections in our single center cohort, the degree of CFTR function may play an important role in development of chronic and mucoid Pa infections and warrants further exploration. Lastly, individuals with worse lung function were more likely to have chronic forms of Pa infection. The observed improvement in lung function during the observation period may also explain the trend in prevalence of both mucoid and chronic Pa.

Restricting the cohort to individuals homozygous for the f508del mutation resulted in a trend, albeit non-significant, toward lower prevalence of both chronic and mucoid Pa. This trend suggests influences aside from change in cohort demographics might be contributing to the change in chronic Pa prevalence. One such influence is the implementation of inhaled anti-pseudomonal antibiotics. Multiple prospective studies involving the use of inhaled and oral anti-pseudomonal antibiotics have demonstrated clearance of newly acquired Pa from respiratory culture [17, 18, 26]. Eradication of this form of Pa from the airways has been associated with improved lung function, fewer pulmonary exacerbations and improved survival [19, 27]. Eradicating newly acquired Pa with inhaled antibiotics is now the standard of care [28]. In the pediatric population, the practice of inhaled anti-pseudomonal antibiotics to eradicate new acquisition of Pa has been associated with a decline in prevalence of chronic Pa infection [29–31]. Whether the attempts to eradicate newly acquired Pa from the airways has translated into a reduction in the frequency of mucoid and chronic Pa in adults with CF is unknown. Our study is the first to analyze change in prevalence of mucoid and chronic Pa in CF adults.

Knowledge about the factors associated with chronic Pa can improve care in CF adults. Identifying risk factors for development of chronic Pa can tailor care and ultimately improve quality of life. In a prospective cohort study, CFTR genotype, female gender, age of diagnosis, and pancreatic enzyme use were identified as risk factors for initial Pa acquisition in children [32]. Predictors of chronic Pa, however, have not been well described in the literature. The limited literature may in part be due to no universally agreed upon definition for chronic Pa. A variable amount of time may lapse between first acquisition of Pa and chronic Pa status, and aggressive attempts to eradicate newly acquired Pa from the airways have lengthened the time between new acquisition and chronic Pa [30]. This may limit our ability to extrapolate risk factors from newly acquired Pa to chronic Pa. The mucoid phenotype of Pa has been associated with increased morbidity and mortality in CF [12, 13] and has been associated with onset of chronic Pa [33]. In children, gender, lower baseline lung function, genotype and absence of MRSA in sputum culture are risk factors associated with acquisition of the mucoid Pa in respiratory culture [34]. Our study is the first to describe independent predictors of chronic and mucoid Pa infection in the adult CF population in the post-pediatric inhaled tobramycin era. Of the predictors, baseline lung function and being diagnosed with CF as an adult appear to be the predominant predictors not being infected with mucoid or chronic Pa. Being diagnosed with CF as an adult is likely reflective of having a milder phenotype, which may correlate with degree of functional CFTR [24]. Whether the predictors found in our study translate to risk factors for the development of chronic and mucoid Pa still needs to be determined.

There are several limitations to consider with this study. Patients cared for at a single CF center were used to conduct this analysis. This both limits generalizability and the ability to detect smaller but clinically meaningful changes in prevalence. Due to challenges eradicating chronic Pa from the airways, a longer observation period may be needed to detect a significant change in the prevalence. To increase the number of observations within each time period, a sensitivity analysis was performed using only two time periods (2002–2007 and 2008–2012). This analysis showed a similar trend toward decreasing prevalence but statistical significance was not met. Incidence of developing chronic Pa may be a better measurement of change, as it would not be influenced by the challenges of eradicating chronic Pa. Our definition of chronic Pa used a modified form of the Leeds criteria and relied on the number of cultures obtained within a calendar year. Under the Leeds definition, chronic Pa was defined as having respiratory cultures positive for Pa in more than 50% of the months within a 12 month period. Collecting respiratory cultures at least every 3 months was recommended when applying this definition [22]. In our adult cohort, the frequency of respiratory cultures on average was less than quarterly. Application of the Leeds definition could have resulted in significant misclassification due too few cultures collected during the year. To reduce misclassification, the analysis restricted the cohort to only include individuals with two or more cultures in a calendar year. This restriction reduced the number of observations available for analysis, which further limited the study’s ability to detect a significant change in prevalence. In our sensitivity analysis using all subjects irrespective of annual culture numbers, a significant decrease in the prevalence of chronic Pa was observed even after adjusting for other variables. This observation suggests that in a larger cohort the trend observed may be significant.

## Conclusion

This analysis is one of the first in recent times to identify independent predictors of mucoid and chronic Pa infection in CF adults and analyze how the prevalence of both mucoid and chronic Pa infection in CF adults have changed with time. What appears to be a decrease in the frequency of chronic and mucoid Pa in recent years warrants further study at a national level to confirm these regional trends and their significance.

## Additional file

Additional file 1: VELVET: list of study investigators and central ethics committees. (DOCX 21 kb)

## Abbreviations

CF: Cystic Fibrosis
CFRD: Cystic fibrosis related diabetes
CFTR: Cystic fibrosis transmembrane conductance regulator
FEV_1_: Forced expiratory volume in one second
GEE: Generalized estimating equation
Pa: *Pseudomonas aeruginosa*
PI: Pancreatic insufficiency
