# Supplementary material for: Change in Pseudomonas aeruginosa prevalence in cystic fibrosis adults over time
Source: BMC Pulm Med. 2016 Dec 7;16:176. doi: 10.1186/s12890-016-0333-y (PMC5142409; doi:10.1186/s12890-016-0333-y)
Supplement: Additional file 1: — VELVET: list of study investigators and central ethics committees. (DOCX 21 kb) [file 12890_2016_333_MOESM1_ESM.docx]

Appendix

VELVET study investigators

Brazil (S Costa, R Hegg, M Mano, J Nunes); Denmark (M Andersson, AH Jacobsen, S Linnet); France (S Delaloge, J Grenier, A-C Hardy Bessard, C Levy, J Medioni, C Moldovan, M Mousseau, T Petit, J-P Spano, B You, L Zelek); Germany (W Abenhardt, S Bauer, T Decker, B Flath, S Fuxius, R Hansen, E Ladda, L Mueller, S Roesel, C Salat, C Schulte, U Soeling, H Tesch, H-W Tessen, J Wamhoff, J Wiegand, M Zaiss); Italy (A Bottini, M Cazzaniga, L Del Mastro, A Falcone, S Gori, P Marchetti, F Roila, S Spazzapan, C Zamagni); Spain (N Batista Lopez, E Blanco Campanario, Y Fernandez Perez, I Garau Llinas, L Gonzalez Cortijo, JM Lopez Vega, R Perez Carrion, A Santaballa Bertran); USA (K Blackwell, H Burstein, V Hansen, RA Hirsch, R Livingston, C Ma, E Macrae, E Mrozek, RH Oyola, G Rodriguez, G Sledge, J Specht, E Tan-Chiu, C Vaughn, C Vogel).

Ethical approval: List of independent ethics committees (IECs) or institutional review boards (IRBs)

Country	Central Ethic Committee
Denmark	The Ethics Committee in the Capital Region of Denmark. Committee E
France	Comité de Protection des Personnes Est IV<br>Hôpitaux Universitaire de Strasbourg<br>1, Place de l'Hôpital<br>67091 STRASBOURG
Germany	Ethik-Kommission der<br>Landesärztekammer Baden-Württemberg<br>Jahnstraße 40<br>70597 Stuttgart
Italy	Comitato Etico Indipendente<br>A.O.U. Policlinico S. Orsola-Malpighi<br>Via Albertoni, 15<br>40138 Bologna
Italy	Comitato Etico Area Vasta Nord Ovest<br>A.O.U. Pisana<br>Via Roma 67<br>56126 PISA PI
Italy	Comitato Etico delle Province di Cremona, Mantova e Lodi<br>V.le Concordia 1<br>26100 Cremona
Italy	Comitato Etico per le Sperimentazioni Cliniche delle Province di Verona e Rovigo<br>Piazzale A. Stefani, 1<br>37126 VERONA

**Country**	**Central Ethic Committee**
Italy	Comitato Etico Regione Liguria - Sez. 2<br>c/o IRCCS A.O. Universitaria S. Martino – IST Istituto Nazionale per la Ricerca sul Cancro di Genova<br>Largo Rosanna Benzi, 10<br>16132 Genova
Italy	Comitato Etico Indipendente<br>CRO IRCCS<br>Via Franco Gallini, 2<br>33081 Aviano
Italy	Comitato Etico delle Aziende Sanitarie dell'Umbria<br>Via della Rivoluzione, 16<br>06070 Ellera di Corciano
Italy	Comitato Etico della Provincia di Monza e Brianza<br>c/o Uff. Sperimentazioni Cliniche<br>A.O. San Gerardo -Villa Serena – III piano<br>Via Pergolesi, 33<br>20900 Monza
Italy	Comitato Etico dell'Università "Sapienza' (Policlinico Universitario Umberto I – A.O. S. Andrea)<br>Viale del Policlinico, 155<br>00161 Roma
Spain	HOSPITAL UNIVERSITARIO CENTRAL DE ASTURIAS<br>Comité Ético de Investigación Clínica. Centro de Rehabilitación - 5ª Planta<br>C/ CELESTINO VILLAMIL, S/N<br>33006- Oviedo
Spain	Comité de Ética de la Investigación de las Illes Balears<br>Consejería de SaludDirección<br>C/ Jesús, 38A Palma de Mallorca 07010
Spain	CEIC del Hospital Universitario de Canarias<br>C/ Ofra, s/n<br>La Cuesta. La Laguna 38320
Spain	Secretaría Técnica del CEIC de Cantabria<br>Fundación Marques de Valdecilla<br>Edificio IFIMAV, 3ª Planta<br>Avda. Cardenal Herrera Oria, s/n<br>39011 Santander
Spain	CEIC Hospital Infanta Cristina Departamento de Farmacología de la Facultad de Medicina<br>Avda. de Elvas, s/n<br>Badajoz 0600
Spain	CEIC del Hospital Puerta de Hierro Secretaria Tecnica del CEIC Planta 1ª - Pasillo Unidades Administrativas (peines 6-7)<br>C\ Joaquín Rodrigo, 2 (Entrada por Laboratorios-Banco de Sangre)<br>28222 Majadahonda
Spain	CEIC Hospital Universitario La Fe<br>Torre A planta 7ª Bulevar Sur, s/n<br>46026 Valencia

Country	Central Ethic Committee
USA	Quorum Review IRB
USA	Washington University in St. Louis Institutional Review Board
USA	Western IRB
USA	Duke University Health System IRB for Clinical Investigations
USA	University of Miami Human Subject Research Office
USA	Mary Washington Healthcare IRB
USA	Dana Farber Cancer Institute IRB
USA	Stanford University Administrative Panel on Human Subjects in Medical Research (multiple rosters)
